# Assessment of the impact of post-processing approaches on absolute myocardial blood flow quantification using cardiac-dedicated CZT-SPECT: A retrospective observational study

**DOI:** 10.1097/MD.0000000000048852

**Published:** 2026-05-15

**Authors:** Yudong Shi, Fukai Zhao, Zekun Pang, Yue Chen, Jiao Wang, Jianming Li

**Affiliations:** a Department of Nuclear Medicine, TEDA International Cardiovascular Hospital, Tianjin University, Tianjin, China.

**Keywords:** absolute myocardial blood flow quantification, cadmium-zinc-telluride, myocardial perfusion imaging, post-processing, single-photon emission computed tomography

## Abstract

Quantitative myocardial blood flow (MBF) assessment using cardiac-dedicated cadmium-zinc-telluride (CZT) single photon emission computed tomography (SPECT) has emerged as a promising noninvasive approach for evaluating both macrovascular and microvascular coronary function. However, the reliability and comparability of MBF measurements are substantially influenced by variations in post-processing methodologies, including the application of attenuation correction (AC) and scatter correction (SC) techniques. Despite the increasing clinical adoption of CZT-SPECT for absolute perfusion quantification, systematic evaluations comparing different post-processing strategies remain limited. This study aimed to evaluate the impact of various post-processing approaches on absolute quantitative MBF measurements obtained through CZT-SPECT, with particular focus on AC and SC methodologies. Seventy patients who underwent dynamic CZT-SPECT myocardial perfusion imaging were retrospectively included, comprising 35 Tc-99m-methoxyisobutylisonitrile patients and 35 Tc-99m-tetrofosmin patients. Each raw imaging dataset was independently reprocessed using Corridor 4DM under 3 correction strategies – no correction, attenuation correction, and attenuation + scatter correction (ACSC) – as well as using MyoFlowQ. Repeated-measures analysis of variance or Friedman tests with Holm-adjusted pairwise comparisons were used to compare the Corridor 4DM processing strategies, and agreement between Corridor 4DM with ACSC and MyoFlowQ was assessed using Bland–Altman analysis. Rest and stress MBF differed significantly across the Corridor 4DM processing strategies (both *P* < .001). Myocardial flow reserve (MFR) differences were tracer-dependent: no overall difference was observed in global left ventricular values in the Tc-99m-methoxyisobutylisonitrile cohort (*P* = .121), whereas a significant difference was observed in the Tc-99m-tetrofosmin cohort (*P* = .002). Compared with Corridor 4DM using ACSC, MyoFlowQ yielded higher stress MBF and MFR values (bias, 1.19–1.31 mL·g^−1^·min^−1^; 95% limits of agreement, –0.30 to 2.68), with moderate inter-software correlation (*r* = 0.44–0.59, *P* < .01). Post-processing methodology significantly influenced absolute MBF quantification in CZT-SPECT imaging. Within Corridor 4DM, correction strategies affected MBF estimates, and MyoFlowQ yielded higher stress MBF and MFR values than Corridor 4DM using ACSC, indicating that these post-processing approaches are not directly interchangeable.

## 1. Introduction

Coronary artery functional imaging techniques have become critical in the diagnosis of myocardial ischemia and treatment decision-making, particularly in noninvasive approaches.^[1]^ Coronary flow reserve (CFR), also known as myocardial flow reserve (MFR), based on quantitative assessment of myocardial blood flow (MBF), represents a powerful tool for evaluating both macrovascular and microvascular coronary function. This measure holds significant clinical utility in the diagnosis, treatment planning, efficacy evaluation, and prognostication of coronary artery disease.^[2]^ Historically, positron emission tomography (PET) has been the sole noninvasive modality for assessing CFR quantification.^[3]^ However, in recent years, cardiac-dedicated cadmium-zinc-telluride (CZT) single photon emission computed tomography (SPECT) dynamic myocardial perfusion imaging (MPI) has emerged as a novel imaging modality that enables absolute quantification of MBF. These CZT-SPECT-based dynamic MFR measurements demonstrate strong correlations with those obtained from PET-derived techniques.^[4]^ In clinical CZT-SPECT MPI, Tc-99m-methoxyisobutylisonitrile (Tc-99m-MIBI) and Tc-99m-tetrofosmin (Tc-99m-TF) are 2 commonly used perfusion tracers. Although both are technetium-99m-labeled agents used for myocardial perfusion assessment, potential differences in tracer behavior may contribute to variability in dynamic quantitative measurements and therefore merit consideration when interpreting software-dependent MBF and MFR results.^[5,6]^ Nevertheless, the post-processing of dynamic MPI data derived from CZT-SPECT systems involves multiple complex steps, including decisions regarding attenuation correction (AC) and/or scatter correction (SC). Variations in these procedural steps, coupled with differences in post-processing software, can significantly impact the final CFR or MFR measurements.^[7–9]^ Previous studies have demonstrated that AC significantly affects MFR measurements,^[10,11]^ yet there remains a lack of systematic investigations in this area. The present study aimed to address this gap by retrospectively reanalyzing quantitative data using distinct post-processing methodologies to investigate the effects of different approaches on absolute MBF quantification as measured by cardiac-dedicated CZT-SPECT dynamic MPI and to assess the inter-method correlations.

## 2. Patients and methods

### 2.1. Patient selection

As this was an exploratory retrospective methodological study, no formal a priori sample size calculation was performed. The study period was restricted to January through March 2024 because, during this interval, our department followed a stable weekly workflow in which dynamic CZT-SPECT studies were scheduled in 2 batches using Tc-99m-MIBI and Tc-99m-TF, respectively. To facilitate a balanced tracer-specific comparison of software-dependent post-processing differences while minimizing temporal heterogeneity in clinical workflow, we included 70 unique patients, comprising 35 Tc-99m-MIBI cases and 35 Tc-99m-TF cases. No patient underwent repeat examination during the study period. These examinations were performed as part of routine clinical care for patients with suspected or known coronary artery disease, including evaluation of ischemic symptoms and, in some cases, follow-up after prior coronary intervention. The study complied with the Declaration of Helsinki and was reviewed by the institutional ethics committee, which determined that the project was exempt from formal approval because only de-identified retrospective data were analyzed. Written informed consent for clinical imaging and secondary research use of de-identified data was obtained from all participants at the time of care.

### 2.2. Imaging equipment, imaging agents, and patient preparation

The Discovery NM 530c cardiac-dedicated CZT-SPECT (NM530c, GE Healthcare) was used, and 99m-TcO_4−_ was supplied by Beijing Atomic High-tech Co., Ltd. or Beijing Senke Co., Ltd. MIBI was supplied by Jiangsu Atomic Medicine Research Institute Jiangyuan Pharmaceutical Factory. TF was supplied by Nanjing Jiangyuan Andiko Positron Research and Development Co., Ltd. Both Tc-99m-MIBI and Tc-99m-TF had radiochemical purity >95%. Within 24 hours prior to imaging, all patients were instructed to avoid any beverages or foods containing caffeine or theobromine and to discontinue vasodilator and calcium channel blocker medications.

### 2.3. Dynamic MPI

Initially, 18.5 to 37.0 MBq of Tc-99m-MIBI or Tc-99m-TF was injected for cardiac positioning. The crosshairs of the anterior, left anterior oblique, and left lateral views were manually adjusted to align with the geometric center of the left ventricle (LV). Following this, rest dynamic acquisition was initiated, and 111 to 185 MBq of Tc-99m-MIBI or Tc-99m-TF was injected after 10 to 15 seconds. After the resting dynamic acquisition was completed, stress dynamic imaging was immediately performed. The stress protocol used was the standard adenosine 6-minute protocol (dosage and intravenous infusion rate at 0.14 mg kg^−1^ min^−1^). At the peak of the stress phase (3 minutes after drug infusion), 388.5 to 647.5 MBq of Tc-99m-MIBI or Tc-99m-TF were injected for stress dynamic imaging (with the stress injection dose at least 3.5 times that of the resting dose). Both acquisitions were conducted in dynamic frame mode, continuously collecting for 10 minutes.^[12]^

### 2.4. Acquisition for CT AC data

All patients underwent a low-dose CT scan (Discovery Elite PET/CT, GE Healthcare) for tissue AC. Acquisition parameters were as follows: tube voltage 120 kV, tube current 20 mA. The patient’s arms were raised, and the scan range covered from the neck to the superior border of the liver. The scan duration was 10.44 seconds, with a slice thickness of 3 mm, and patients were instructed to breathe calmly during the acquisition.

### 2.5. Quantitative blood flow processing using MyoFlowQ software

At our institution, routine clinical post-processing of dynamic CZT-SPECT MPI was performed using MyoFlowQ. For the present retrospective study, the same raw imaging datasets were additionally reprocessed offline using Corridor 4DM under different correction strategies for comparative analysis. The 2 sets of dynamic data and CT AC data were transferred to the processing workstation MyoFlowQ 1.0 version (Beijing Bailing Cloud Medical Technology Co., Ltd.) for comprehensive physical correction, including AC, SC, and spatial-resolution recovery, followed by image reconstruction.^[13]^ A single-tissue, 2-compartment kinetic model was applied to derive kinetic parameters from the myocardial and arterial input time-activity curves, which were then converted to MBF expressed in mL·g^−1^·min^−1^. Quantitative parameters included resting MBF (rMBF), stress MBF (sMBF), and MFR calculated as MFR = sMBF/rMBF for the left anterior descending artery (LAD), left circumflex artery (LCX), right coronary artery (RCA), and the global LV.

### 2.6. Quantitative blood flow processing using Corridor 4DM software

The 2 sets of dynamic and CT AC data were transferred to the processing workstation Xeleris 4DR (GE Healthcare). The Lister tool was first used to process the 2 sets of dynamic data without AC (no correction [NC]), with AC, and with attenuation correction + scatter correction (ACSC) to obtain 3 sets of data for blood flow quantification. Then, the specialized software Corridor 4DM 2017 version (INVIA) was used to post-process the 3 sets of data for blood flow quantification. Single-compartment kinetic and net retention models were applied to estimate myocardial uptake and myocardial retention rates, which were then converted to MBF expressed in mL·g^−1^·min^−1^. The final quantitative parameters (rMBF, sMBF, MFR for LAD/LCX/RCA and global LV) were exported from Corridor 4DM, with MFR = sMBF/rMBF.

### 2.7. Statistical analysis

Quantitative variables were expressed as mean ± standard deviation (SD). Normality of paired differences was assessed with the Shapiro–Wilk test; sphericity was examined by Mauchly test when applicable. Within-subject comparisons across the three 4DM algorithms (NC/AC/ACSC) used repeated-measures analysis of variance (with Greenhouse–Geisser correction when sphericity was violated) or the Friedman test when assumptions were not met, followed by Holm-adjusted pairwise comparisons (paired *t* tests or Wilcoxon signed-rank tests, as appropriate). Agreement between 4DM-ACSC and MyoFlowQ was evaluated with Bland–Altman analysis, reporting bias and 95% limits of agreement (LOA = bias ± 1.96 SD) and testing for proportional bias by regressing the difference on the mean. Linear associations were assessed with Pearson correlation. Two-sided adjusted *P* < .05 was considered statistically significant.

## 3. Results

### 3.1. Patient characteristics

A total of 70 patients were enrolled (35 Tc-99m-MIBI and 35 Tc-99m-TF; Table 1). Most patients presented with atypical chest pain (81.4%). Hypertension was the most prevalent risk factor (71.4%), followed by hyperlipidemia (42.9%) and diabetes mellitus (~20%). A history of percutaneous coronary intervention was present in 22.9% of patients.

**Table 1:** Demographic and clinical characteristics of the study population.

Variable	Overall (n = 70)	99mTc-MIBI group (n = 35)	99mTc-TF group (n = 35)	Statistic	*P* value
Age (yr)	59.7 ± 10.7	60.0 ± 10.4	59.4 ± 11.2	0.233	.817
Sex, n (%)				2.100	.147
Male	30 (42.9)	12 (34.3)	18 (51.4)		
Female	40 (57.1)	23 (65.7)	17 (48.6)		
Height (cm)	1.64 ± 0.08	1.63 ± 0.09	1.64 ± 0.08	−1.450	.061
Weight (kg)	72.9 ± 11.4	74.0 ± 13.5	72.0 ± 8.9	1.450	.061
BMI (kg/m^2^)	26.8 ± 4.01	27.5 ± 4.2	26.1 ± 3.7	−0.366	.716
Symptoms, n (%)				0.736	.464
Typical angina	5 (7.1)	3 (8.6)	2 (5.7)		
Atypical angina	57 (81.4)	28 (80.0)	30 (85.7)		
Non-anginal chest pain	8 (11.4)	4 (11.4)	3 (8.6)		
Hypertension, n (%)	50 (71.4)	25 (71.4)	25 (71.4)	0.412	.814
Hyperlipidemia, n (%)	30 (42.9)	16 (45.7)	14 (40.0)	0.458	.360
Diabetes mellitus, n (%)	14 (20.0)	4 (11.4)	10 (28.6)	−0.627	.211
Smoking, n (%)	20 (28.6)	6 (17.1)	14 (40.0)	0.393	.433
Alcohol use, n (%)	12 (17.1)	6 (17.1)	6 (17.1)	0.000	.629
Family history, n (%)	8 (11.4)	6 (17.1)	2 (5.7)	0.233	.073
Prior PCI, n (%)	16 (22.9)	10 (28.6)	6 (17.1)	3.214	.034
Prior MI, n (%)	1 (1.4)	1 (2.9)	0 (0.0)	4.480	1.000
Resting heart rate (bpm)	69 ± 11	70 ± 11	68 ± 11	2.258	.133
Resting blood pressure (mm Hg)					
SBP	124 ± 17	123 ± 18	124 ± 17	−0.357	.723
DBP	71 ± 10	71 ± 12	72 ± 9	−0.522	.603

Data are presented as mean ± standard deviation or n (%). Test statistics are reported as *t*, *χ*^2^, or *Z* values, as appropriate.

BMI = body mass index, bpm = beats per minute, DBP = diastolic blood pressure, MI = myocardial infarction, mm Hg = millimeters of mercury, PCI = percutaneous coronary intervention, SBP = systolic blood pressure.

### 3.2. Analysis of quantitative parameters across 3 image processing algorithms in Corridor 4DM

Using the within-subject framework summarized in Table 2, rMBF and sMBF differed significantly across the three 4DM algorithms for both tracers (global tests, all *P* < .001). Post-hoc Holm-adjusted comparisons showed NC > AC and NC > ACSC for rMBF/sMBF. For sMBF, AC < ACSC consistently; for rMBF, AC and ACSC were broadly comparable at the LV level, while territory-level differences were observed (e.g., LCX in the MIBI cohort; see Table 2). In contrast, MFR was tracer and territory dependent. At the LV level, MIBI showed no overall difference (*χ*^2^ = 4.22, *P* = .121), whereas TF did (*χ*^2^ = 12.23, *P* = .002); territory-level differences were also observed (e.g., MIBI-RCA *χ*^2^ = 9.58, *P* = .008; TF-LAD/LCX/RCA all *P* < .05). Across significant contrasts, the common pattern was AC < (NC ≈ ACSC).

**Table 2:** Comparative analysis of 3 post-processing methods for the quantitative assessment of myocardial blood flow using Corridor 4DM software.

Groups	Quantitative values	Area	Corridor 4DM (mean ± SD)			Global test (*χ*^2^/*F*; *P*)	Pairwise comparisons (Holm-adjusted *P*; *W*/*t*)		
			NC	AC	ACSC		NC vs AC	NC vs ACSC	AC vs ACSC
Tc-99m-MIBI group	rMBF	LAD	1.34 ± 0.62	0.71 ± 0.30	0.76 ± 0.35	*χ*^2^ = 55.01; *P* < .001*	*<.001; *W* = 0.00	*<.001; *W* = 0.00	.072; *W* = 180.00
		LCX	1.20 ± 0.57	0.79 ± 0.33	0.96 ± 0.56	*χ*^2^ = 43.64; *P* < .001*	*<.001; *W* = 0.00	*<.001; *W* = 67.50	*.005; *W* = 113.50
		RCA	1.13 ± 0.50	0.77 ± 0.32	0.86 ± 0.56	*χ*^2^ = 44.23; *P* < .001*	*<.001; *W* = 0.00	*<.001; *W* = 67.50	.198; *W* = 208.50
		LV	1.19 ± 0.55	0.72 ± 0.30	0.75 ± 0.36	*χ*^2^ = 54.32; *P* < .001*	*<.001; *W* = 0.00	*<.001; *W* = 0.00	.266; *W* = 232.50
	sMBF	LAD	1.69 ± 0.72	0.82 ± 0.37	1.00 ± 0.54	*χ*^2^ = 55.71; *P* < .001*	*<.001; *W* = 0.00	*<.001; *W* = 0.00	*.005; *W* = 145.50
		LCX	1.70 ± 0.80	0.98 ± 0.46	1.31 ± 0.79	*χ*^2^ = 41.19; *P* < .001*	*<.001; *W* = 6.50	*<.001; *W* = 82.00	*<.001; *W* = 62.50
		RCA	1.79 ± 0.66	1.04 ± 0.48	1.33 ± 0.81	*χ*^2^ = 42.06; *P* < .001*	*<.001; *W* = 0.00	*<.001; *W* = 69.00	*.001; *W* = 109.50
		LV	1.66 ± 0.70	0.89 ± 0.40	1.10 ± 0.64	*χ*^2^ = 50.69; *P* < .001*	*<.001; *W* = 0.00	*<.001; *W* = 11.00	*.003; *W* = 79.50
	MFR	LAD	1.40 ± 0.75	1.25 ± 0.64	1.35 ± 0.74	*χ*^2^ = 2.23; *P* = .328	.226; *W* = 205.50	.545; *W* = 247.00	.545; *W* = 271.00
		LCX	1.58 ± 0.82	1.37 ± 0.77	1.58 ± 0.99	*χ*^2^ = 5.89; *P* = .053	.052; *W* = 171.00	.605; *W* = 251.00	.605; *W* = 261.00
		RCA	1.74 ± 0.85	1.47 ± 0.82	1.77 ± 1.08	*χ*^2^ = 9.58; *P* = .008*	*.001; *W* = 90.00	.501; *W* = 272.50	.470; *W* = 228.00
		LV	1.53 ± 0.78	1.34 ± 0.72	1.53 ± 0.88	*χ*^2^ = 4.22; *P* = .121	*.021; *W* = 139.50	.602; *W* = 265.50	.602; *W* = 237.00
Tc-99m-TF group	rMBF	LAD	1.06 ± 0.46	0.56 ± 0.20	0.61 ± 0.28	*χ*^2^ = 54.43; *P* < .001*	*<.001; *W* = 0.00	*<.001; *W* = 0.00	.081; *W* = 183.00
		LCX	1.04 ± 0.49	0.66 ± 0.29	0.77 ± 0.45	*χ*^2^ = 45.31; *P* < .001*	*<.001; *W* = 0.00	*<.001; *W* = 25.00	*.006; *W* = 151.00
		RCA	1.03 ± 0.43	0.67 ± 0.26	0.72 ± 0.39	*χ*^2^ = 42.91; *P* < .001*	*<.001; *W* = 2.00	*<.001; *W* = 28.00	.127; *W* = 220.50
		LV	1.00 ± 0.44	0.59 ± 0.23	0.63 ± 0.32	*χ*^2^ = 53.11; *P* < .001*	*<.001; *W* = 0.00	*<.001; *W* = 0.00	.146; *W* = 212.50
	sMBF	LAD	1.50 ± 0.80	0.68 ± 0.26	0.76 ± 0.32	*χ*^2^ = 55.71; *P* < .001*	*<.001; *W* = 0.00	*<.001; *W* = 0.00	*.012; *W* = 139.50
		LCX	1.61 ± 0.78	0.84 ± 0.34	1.09 ± 0.49	*χ*^2^ = 45.51; *P* < .001*	*<.001; *W* = 1.00	*<.001; *W* = 26.00	*<.001; *W* = 56.00
		RCA	1.70 ± 0.77	0.92 ± 0.31	1.05 ± 0.35	*χ*^2^ = 42.23; *P* < .001*	*<.001; *W* = 4.00	*<.001; *W* = 22.00	*<.001; *W* = 116.50
		LV	1.51 ± 0.75	0.75 ± 0.28	0.86 ± 0.34	*χ*^2^ = 53.23; *P* < .001*	*<.001; *W* = 0.00	*<.001; *W* = 2.00	*.003; *W* = 122.50
	MFR	LAD	1.53 ± 0.74	1.28 ± 0.48	1.44 ± 0.75	*χ*^2^ = 7.71; *P* = .021*	*.002; *W* = 90.50	.477; *W* = 253.50	.477; *W* = 242.00
		LCX	1.73 ± 0.85	1.42 ± 0.60	1.75 ± 1.09	*χ*^2^ = 10.43; *P* = .005*	*<.001; *W* = 62.00	.827; *W* = 301.00	*.023; *W* = 128.50
		RCA	1.76 ± 0.65	1.49 ± 0.57	1.74 ± 0.84	*F* = 7.39; *P* = .001*	*<.001; *t* = 5.42	.871; *t* = 0.16	*.011; *t* = −2.97
		LV	1.64 ± 0.73	1.37 ± 0.51	1.61 ± 0.81	*χ*^2^ = 12.23; *P* = .002*	*<.001; *W* = 70.50	.680; *W* = 288.50	*.036; *W* = 148.00

AC = attenuation correction, ACSC = attenuation correction + scatter correction, LAD = left anterior descending artery, LCX = left circumflex artery, LV = left ventricle, MFR = myocardial flow reserve, NC = no correction, RCA = right coronary artery, rMBF = rest myocardial blood flow, sMBF = stress myocardial blood flow.

* Data are presented as mean ± standard deviation. rMBF and sMBF are expressed in mL g^−1^ min^−1^; MFR is unitless. Global comparisons across NC, AC, and ACSC were performed using repeated-measures analysis of variance (*F*) or the Friedman test (*χ*^2^), as appropriate. Pairwise comparisons were performed using paired *t* tests (*t*) or Wilcoxon signed-rank tests (*W*), with Holm adjustment for multiple comparisons. Adjusted *P* < .05.

For illustration at the LV level in the MIBI cohort, means (±SD, mL·g^−1^·min^−1^) were 1.19 ± 0.55/0.72 ± 0.30/0.75 ± 0.36 for rMBF and 1.66 ± 0.70/0.89 ± 0.40/1.10 ± 0.64 for sMBF (NC/AC/ACSC), whereas MFR was 1.53 ± 0.78/1.34 ± 0.72/1.53 ± 0.88; NC vs AC was significant (adjusted *P* = .021), but NC vs ACSC was not (adjusted *P* = .602). For TF at the LV level, NC vs AC remained significant (adjusted *P* < .001), NC vs ACSC was not (*P* = .680), and AC vs ACSC reached significance (adjusted *P* = .036), again indicating AC < (NC ≈ ACSC).

### 3.3. Consistency comparison between post-processing methods in Corridor 4DM and MyoFlowQ

**Figure 1.**
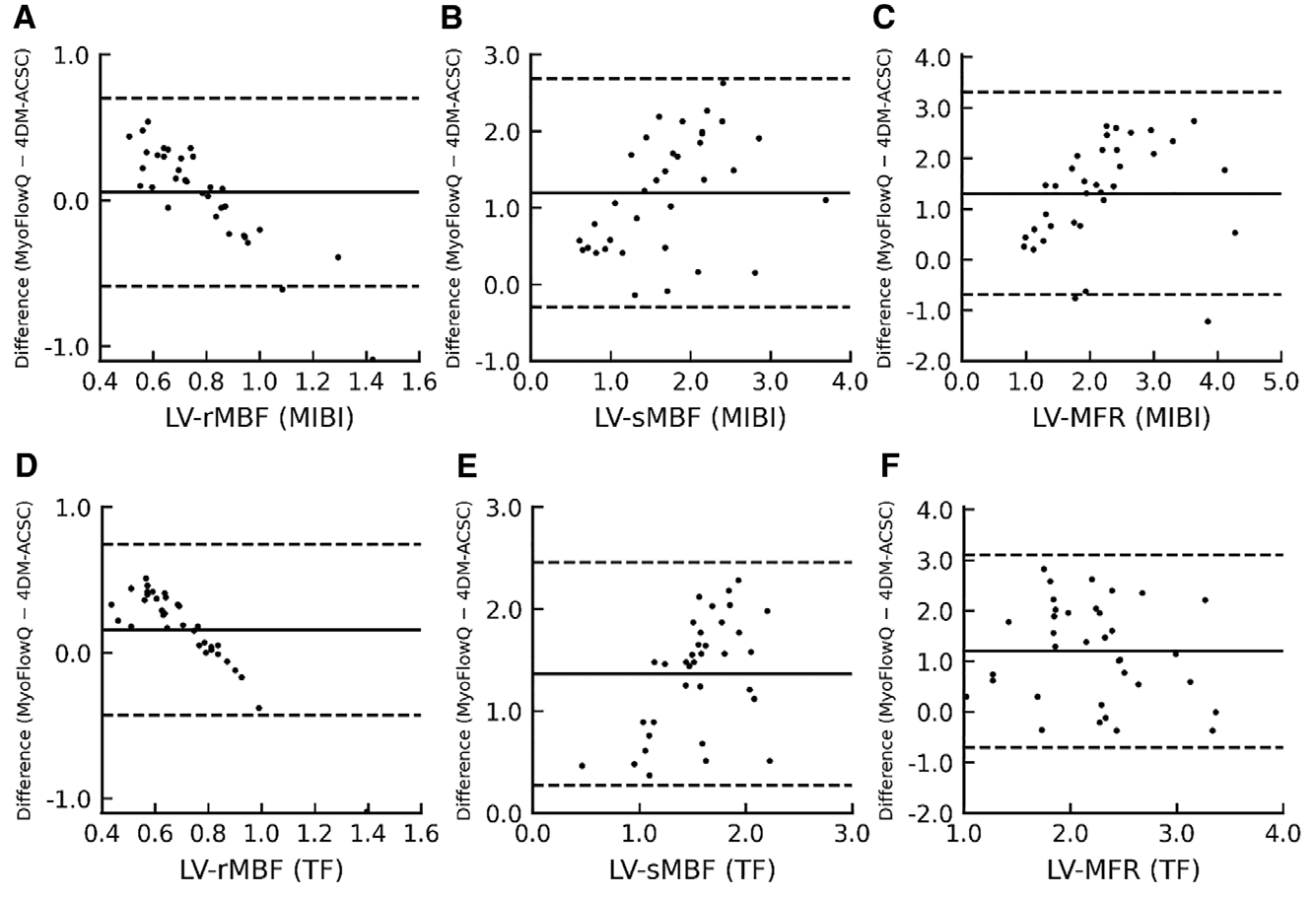
Bland–Altman plots comparing MyoFlowQ and 4DM-ACSC for LV perfusion parameters. (A–C) Tc-99m-MIBI group. (D–F) Tc-99m-TF group. ACSC = attenuation correction + scatter correction, LV = left ventricle, Tc-99m-MIBI = technetium-99m-methoxyisobutylisonitrile, Tc-99m-TF = technetium-99m-tetrofosmin.

Bland–Altman analysis (bias defined as MyoFlowQ – 4DM-ACSC) indicated small bias for LV rMBF with relatively narrow 95% LOA: MIBI, bias 0.06 with LOA −0.59 to 0.70; TF, bias 0.16 with LOA −0.43 to 0.74. In contrast, LV sMBF showed a positive bias with wider LOA (MIBI: 1.19, −0.30 to 2.68; TF: 1.36, 0.27 to 2.46). LV MFR also exhibited a positive bias (MIBI: 1.31, −0.69 to 3.30; TF: 1.20, −0.71 to 3.10). These findings suggest that MyoFlowQ yields higher sMBF and MFR than 4DM-ACSC, whereas rMBF is close on average. Full BA results for all territories are reported in Table 3. The Bland–Altman plots for LV rMBF, sMBF and MFR are shown in Figure 1, demonstrating that MyoFlowQ yields higher sMBF and MFR values than 4DM-ACSC, whereas rMBF shows minimal bias.

**Table 3:** Agreement between Corridor 4DM (NC, AC, ACSC) and MyoFlowQ: Bland–Altman analysis.

Groups	Quantitative values	Territories	Corridor 4DM (mean ± SD)			MyoFlowQ (mean ± SD)	Bland–Altman (bias; 95% LOA)		
			NC	AC	ACSC		NC vs MyoFlowQ	AC vs MyoFlowQ	ACSC vs MyoFlowQ
Tc-99m-MIBI group	rMBF	LAD	1.34 ± 0.62	0.71 ± 0.30	0.76 ± 0.35	0.83 ± 0.07	−0.51 (−1.71/0.69)	0.12 (−0.46/0.70)	0.07 (−0.57/0.70)
		LCX	1.20 ± 0.57	0.79 ± 0.33	0.96 ± 0.56	0.76 ± 0.10	−0.44 (−1.57/0.68)	−0.03 (−0.66/0.59)	−0.20 (−1.24/0.85)
		RCA	1.13 ± 0.50	0.77 ± 0.32	0.86 ± 0.56	0.82 ± 0.14	−0.31 (−1.24/0.62)	0.05 (−0.57/0.68)	−0.04 (−1.10/1.03)
		LV	1.19 ± 0.55	0.72 ± 0.30	0.75 ± 0.36	0.81 ± 0.09	−0.39 (−1.45/0.68)	0.09 (−0.48/0.66)	0.06 (−0.59/0.70)
	sMBF	LAD	1.69 ± 0.72	0.82 ± 0.37	1.00 ± 0.54	2.32 ± 0.92	0.63 (−0.72/1.98)	1.50 (−0.12/3.11)	1.32 (−0.10/2.75)
		LCX	1.70 ± 0.80	0.98 ± 0.46	1.31 ± 0.79	1.96 ± 0.87	0.26 (−0.98/1.50)	0.99 (−0.39/2.36)	0.65 (−0.83/2.13)
		RCA	1.79 ± 0.66	1.04 ± 0.48	1.33 ± 0.81	2.60 ± 1.16	0.81 (−1.06/2.69)	1.56 (−0.52/3.64)	1.27 (−0.85/3.39)
		LV	1.66 ± 0.70	0.89 ± 0.40	1.10 ± 0.64	2.30 ± 0.93	0.63 (−0.68/1.94)	1.41 (−0.18/3.00)	1.19 (−0.30/2.68)
	MFR	LAD	1.40 ± 0.75	1.25 ± 0.64	1.35 ± 0.74	2.79 ± 1.06	1.39 (−0.78/3.57)	1.54 (−0.62/3.70)	1.44 (−0.47/3.35)
		LCX	1.58 ± 0.82	1.37 ± 0.77	1.58 ± 0.99	2.57 ± 1.11	0.99 (−1.14/3.12)	1.20 (−0.95/3.35)	1.00 (−1.16/3.16)
		RCA	1.74 ± 0.85	1.47 ± 0.82	1.77 ± 1.08	3.09 ± 1.25	1.34 (−1.31/3.99)	1.62 (−0.99/4.22)	1.32 (−1.23/3.86)
		LV	1.53 ± 0.78	1.34 ± 0.72	1.53 ± 0.88	2.83 ± 1.12	1.30 (−0.91/3.51)	1.50 (−0.70/3.69)	1.31 (−0.69/3.30)
Tc-99m-TF group	rMBF	LAD	1.06 ± 0.46	0.56 ± 0.20	0.61 ± 0.28	0.82 ± 0.06	−0.24 (−1.11/0.62)	0.26 (−0.10/0.62)	0.21 (−0.30/0.73)
		LCX	1.04 ± 0.49	0.66 ± 0.29	0.77 ± 0.45	0.72 ± 0.10	−0.33 (−1.22/0.57)	0.06 (−0.46/0.58)	−0.06 (−0.87/0.75)
		RCA	1.03 ± 0.43	0.67 ± 0.26	0.72 ± 0.39	0.82 ± 0.10	−0.21 (−0.95/0.54)	0.15 (−0.28/0.58)	0.10 (−0.58/0.79)
		LV	1.00 ± 0.44	0.59 ± 0.23	0.63 ± 0.32	0.79 ± 0.07	−0.22 (−1.01/0.58)	0.20 (−0.20/0.59)	0.16 (−0.43/0.74)
	sMBF	LAD	1.50 ± 0.80	0.68 ± 0.26	0.76 ± 0.32	2.23 ± 0.63	0.72 (−0.82/2.27)	1.55 (0.40/2.70)	1.46 (0.33/2.60)
		LCX	1.61 ± 0.78	0.84 ± 0.34	1.09 ± 0.49	1.90 ± 0.60	0.30 (−1.22/1.81)	1.06 (−0.01/2.13)	0.81 (−0.39/2.02)
		RCA	1.70 ± 0.77	0.92 ± 0.31	1.05 ± 0.35	2.57 ± 0.76	0.87 (−0.77/2.51)	1.65 (0.20/3.10)	1.51 (0.05/2.98)
		LV	1.51 ± 0.75	0.75 ± 0.28	0.86 ± 0.34	2.23 ± 0.59	0.72 (−0.73/2.17)	1.48 (0.41/2.55)	1.36 (0.27/2.46)
	MFR	LAD	1.53 ± 0.74	1.28 ± 0.48	1.44 ± 0.75	2.70 ± 0.73	1.17 (−0.49/2.83)	1.41 (−0.10/2.93)	1.25 (−0.57/3.08)
		LCX	1.73 ± 0.85	1.42 ± 0.60	1.75 ± 1.09	2.64 ± 0.74	0.92 (−1.02/2.85)	1.23 (−0.49/2.95)	0.89 (−1.59/3.37)
		RCA	1.76 ± 0.65	1.49 ± 0.57	1.74 ± 0.84	3.12 ± 0.85	1.36 (−0.48/3.20)	1.63 (−0.18/3.44)	1.38 (−0.86/3.61)
		LV	1.64 ± 0.73	1.37 ± 0.51	1.61 ± 0.81	2.81 ± 0.70	1.17 (−0.46/2.80)	1.44 (−0.08/2.96)	1.20 (−0.71/3.10)

Data are presented as mean ± standard deviation. rMBF and sMBF are expressed in mL·g^−1^·min^−1^; MFR is unitless. Bland–Altman analysis is presented as bias and 95% LOA. Bias was calculated as MyoFlowQ minus Corridor 4DM; a positive bias indicates higher values obtained with MyoFlowQ.

AC = attenuation correction, ACSC = attenuation correction + scatter correction, LAD = left anterior descending artery, LCX = left circumflex artery, LOA = limits of agreement, LV = left ventricle, MFR = myocardial flow reserve, NC = no correction, RCA = right coronary artery, rMBF = rest myocardial blood flow, sMBF = stress myocardial blood flow.

### 3.4. Correlation between quantitative parameters from 4DM-ACSC and MyoFlowQ

**Figure 2.**
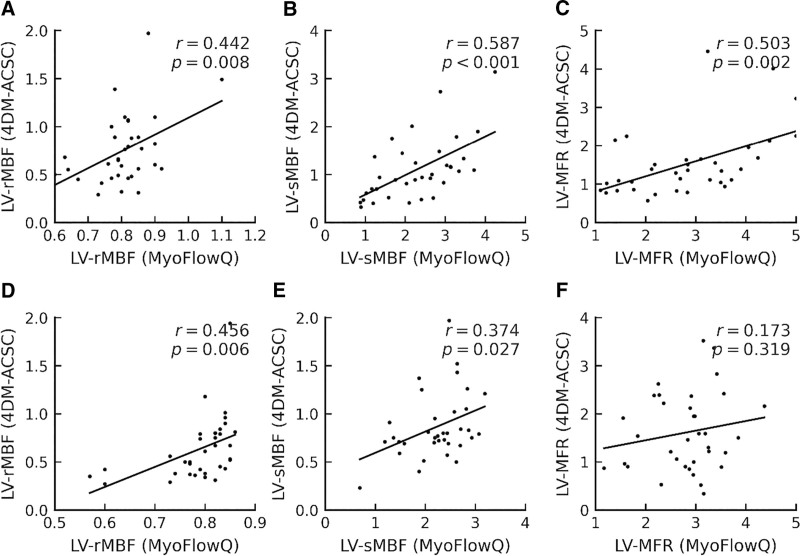
Pearson correlation between the processing results of using Corridor 4DM-ACSC and MyoFlowQ in Tc-99m-MIBI and Tc-99m-TF groups. (A–C) Tc-99m-MIBI group. (D–F) Tc-99m-TF group. ACSC = attenuation correction + scatter correction, Tc-99m-MIBI = technetium-99m-methoxyisobutylisonitrile, Tc-99m-TF = technetium-99m-tetrofosmin.

Figure 2 depicts the Pearson correlation between 4DM-ACSC and MyoFlowQ measurements. In the Tc-99m-MIBI cohort, moderate positive correlations were observed for LV rMBF (*r* = 0.442, *P* = .008), LV sMBF (*r* = 0.587, *P* < .001), and LV MFR (*r* = 0.503, *P* = .002). In the Tc-99m-TF cohort, correlations were *r* = 0.514 (*P* = .002) for LV rMBF, *r* = 0.374 (*P* = .027) for LV sMBF, and not significant for LV MFR (*r* = 0.173, *P* = .319). Because correlation does not imply interchangeability, method agreement was primarily assessed using the Bland–Altman results (Table 3).

## 4. Discussion

Noninvasive absolute MBF quantification holds significant clinical implications, serving as a cornerstone for the diagnosis and management of coronary artery disease.^[14]^ While this technique accurately identifies and evaluates the severity of coronary artery multivessel disease, it also plays a crucial role in detecting coronary microvascular dysfunction and assessing prognosis.^[15]^ However, the variability in post-processing methods for cardiac-dedicated CZT-SPECT myocardial perfusion dynamic acquisition data poses a challenge in achieving consistent and reliable results across studies and clinical applications.^[16]^ The discrepancies in MBF quantification arise from differences in image reconstruction algorithms, AC, and/or SC techniques.^[17,18]^

This study highlights that the presence or absence of AC and/or SC, coupled with the choice of software, significantly impacts the rest and stress MBF calculations. For instance, the absence of AC in software such as Corridor 4DM was found to result in notable differences in MBF values across the coronary artery regions and the entire heart, thereby affecting the MFR. Statistical analysis revealed that quantitative measurements before and after AC exhibited significant differences, underscoring the importance of proper correction techniques. Furthermore, the application of SC in addition to AC introduced additional variability in the results, with significant differences observed in many quantitative parameters between the 2 approaches.

Notably, MFR differences emerged in the TF cohort at the LV level and across territories, whereas MIBI LV showed no overall difference; when present, the pairwise pattern consistently favored AC < (NC ≈ ACSC). In comparing MFR across software platforms, MyoFlowQ – implementing full physical correction (attenuation, scatter, and resolution recovery) – yielded systematically higher MFR than Corridor 4DM. This pattern was consistent in both tracer cohorts and is reflected by positive biases on Bland–Altman analysis, rather than by significance testing. At the same time, rMBF biases were small with relatively narrow LOA, whereas sMBF biases were larger with wider LOA, indicating that method-specific corrections have a differential impact on stress flow and reserve.

These findings should also be interpreted in the context of previous validation studies of dynamic CZT-SPECT. Prior work has shown that CZT-SPECT-derived MBF and MFR are technically feasible and may show reasonable agreement with established reference methods under optimized conditions. For example, the WATERDAY study demonstrated that dynamic Tc-99m-sestamibi CZT-SPECT yielded MFR values comparable to those obtained with ^15^O-water PET and showed good diagnostic performance for functionally significant coronary artery disease.^[4]^ Similarly, Otaki et al reported improved agreement between CZT-SPECT and ^15^O-water PET after motion correction and optimized arterial input function placement.^[19]^ However, interchangeability across modalities is not absolute; Acampa et al found that hyperemic MBF and myocardial perfusion reserve derived from CZT-SPECT could be systematically higher than those obtained with ^82^Rb PET, with only moderate correlation between the 2 techniques.^[3]^ In parallel, technical studies have emphasized that AC and other processing choices materially influence quantitative CZT-SPECT results,^[7]^ and operator-dependent variability has also been reported.^[20]^ Against this background, the present study extends the literature by showing that even when the raw dynamic datasets are identical, differences in post-processing strategy alone can materially alter absolute MBF and derived MFR estimates.

Collectively, these findings reinforce that post-processing choices (AC/SC and software implementation) materially influence absolute MBF quantification and derived MFR. Accordingly, inter-software interchangeability should be judged by Bland–Altman bias and LOA, not by correlation alone. Standardized post-processing protocols and transparent reporting of correction pipelines are essential for reproducibility and multicenter comparability.

Several limitations of this study should be acknowledged. First, this was a single-center exploratory retrospective methodological study with a modest sample size, and no formal a priori sample size calculation was performed. Second, patients with prior percutaneous coronary intervention were not excluded because the aim was to compare post-processing methods using identical raw datasets rather than to assess diagnostic performance in specific clinical subgroups; however, prior revascularization may affect myocardial perfusion characteristics, and no dedicated subgroup analysis was performed. Third, no external reference standard such as PET, invasive FFR, or coronary angiography was available, so we could not determine the superiority or greater accuracy of either Corridor 4DM or MyoFlowQ. Accordingly, our findings should be interpreted as demonstrating software-dependent differences and limited inter-method interchangeability rather than superiority of one platform over the other.

## 5. Conclusion

Post-processing methodology materially influenced absolute MBF and MFR estimates derived from identical dynamic CZT-SPECT datasets. Within Corridor 4DM, correction strategies affected quantitative values, and MyoFlowQ yielded higher stress MBF and MFR values than Corridor 4DM using ACSC. These findings indicate that the 2 post-processing approaches should not be considered directly interchangeable, support the need for standardized and transparently reported post-processing pipelines, and warrant further validation against external reference standards before any inference regarding superiority or accuracy can be made.

## Acknowledgments

The authors used ChatGPT (OpenAI, GPT-4) to assist with English language editing. The authors are fully responsible for the content of this manuscript.

## Author contributions

**Conceptualization:** Yudong Shi.

**Investigation:** Yudong Shi, Zekun Pang.

**Writing – original draft:** Yudong Shi, Fukai Zhao.

**Methodology:** Zekun Pang, Jianming Li.

**Data curation:** Yue Chen, Jiao Wang.

**Formal analysis:** Yue Chen, Jiao Wang.

**Writing – review & editing:** Jiao Wang, Jianming Li.

**Project administration:** Jianming Li.

**Supervision:** Jianming Li.
